# Identification of 19-*epi*-okadaic Acid, a New Diarrhetic Shellfish Poisoning Toxin, by Liquid Chromatography with Mass Spectrometry Detection

**DOI:** 10.3390/md20080024

**Published:** 2008-08-28

**Authors:** Beatriz Paz, Antonio H. Daranas, Patricia G. Cruz, José M. Franco, Manuel Norte, José J. Fernández

**Affiliations:** 1 Fitoplancton Tóxico, Instituto de Investigaciones Marinas (CSIC), Eduardo Cabello 6, 36080 Vigo and Instituto Español de Oceanografía, Centro Oceanográfico de Vigo (IEO), Cabo Estay, 36200 Vigo, Spain. E-mails: beatriz.paz@vi.ieo.es (B. Paz); jose.franco@vi.ieo.es (J.M. Franco); 2 Instituto Universitario de Bio-Orgánica “Antonio González”, Universidad de La Laguna, Astrofísico Francisco Sánchez 2, 38206 La Laguna, Tenerife, Spain. E-mails: adaranas@ull.es (A. Daranas); mnorte@ull.es (M. Norte); jjfercas@ull.es (J.J. Fernández); 3 Departamento de Ingeniería Química y Tecnología Farmacéutica; Universidad de La Laguna; Av. Astrofísico Francisco Sánchez 1, 38071, La Laguna, Tenerife, Spain

**Keywords:** Marine toxins, DSP, 19-*epi*-okadaic acid, *Prorocentrum belizeanum*, LC-MS

## Abstract

Okadaic acid (**1**) (OA) and its congeners are mainly responsible for diarrhetic shellfish poisoning (DSP) syndrome. The presence of several OA derivatives have already been confirmed in *Prorocentrum* and *Dinophysis* spp. In this paper, we report on the detection and identification of a new DSP toxin, the OA isomer 19-*epi*-okadaic acid (**2**) (19-*epi*-OA), isolated from cultures of *Prorocentrum belizeanum*, by determining its retention time (RT) and fragmentation pattern using liquid chromatography coupled with mass spectrometry (LC–MS/MS).

## 1. Introduction

Okadaic acid (**1**) (OA) and dinophysistoxins (DTXs) are the main toxins responsible for a severe gastrointestinal illness, also known as diarrhetic shellfish poisoning syndrome (DSP), which results from the consumption of marine seafood contaminated with toxigenic dinoflagellates of the genus *Dinophysis* or *Prorocentrum* [1,2]. Due to its significance to public health and the economy of the fishing industry, many monitoring and control programmes have been implemented [3, 4]. Over the last decades, the use of chemical methods to detect these toxins have become routine, the most important ones used being liquid chromatography using fluorometric detection (LC-FLD) of anthryldiazomethane (ADAM) derivatives [5], and liquid chromatography coupled with mass spectrometry (LC-MS) [6,7]. In spite of the high cost of the equipment, LC-MS technique has been successful in control laboratories because of the wealth of information it provides, as well as for its versatility, since it can be used to detect many different types of marine toxins [8]. Therefore, considerable advances to increase the number of targets for the analysis of contaminated shellfish have been made, especially since the use of extracts obtained from cultures of dinoflagellates were introduced [9–11].

Interestingly, it has been reported in the analyses of DSP toxins by different authors, that in addition to the peaks of OA, DTXs, and their ester derivatives, other unidentified peaks were detected [12,13]. Based on the fact that these peaks gave the same fragmentation pattern observed for OA when collision-induced dissociation was used, the authors suggested that they correspond to OA isomers [7]. In the first of these articles, one of the new compounds eluted before OA with a retention time very close, but not identical, to DTX2 [12]. Recently an article providing detailed profiles of 7-*O*-acyl ester in plankton and shellfish from the Portuguese coast has been published and similar findings were again reported [13].

## 2. Results and Discussion

**Figure 2:**
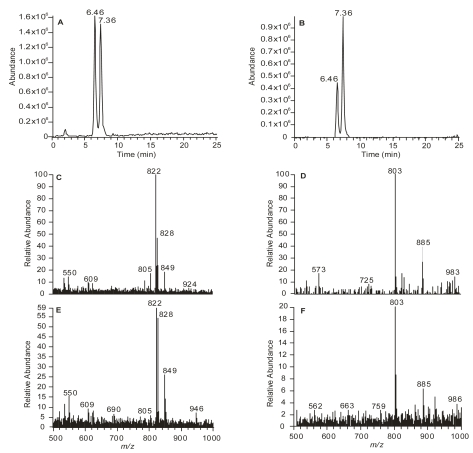
LC-MS ion chromatograms for both 19-*epi*-okadaic acid (**2**) at RT 6.46 min and okadaic acid (**1**) at RT 7.36 min, (A) in positive ionization mode at *m*/*z* 822 [M+NH_4_]^+^ and (B) in negative ionization mode at *m*/*z* 803 [M−H]^−^. Positive and negative mass spectra for the okadaic acid (**1**) (C and D); and for the 19-*epi*-okadaic acid (**2**) (E and F).

The isolation and structural determination of a new DSP toxin derivative called 19-*epi*-okadaic acid, (**2**) (19-*ep*i-OA) from cultures of the dinoflagellate *Prorocentrum belizeanum* [14,15], prompted us to undertake an analytical study of this metabolite. A full characterization of this new molecule in our laboratory based on the use of NMR, MS and computational techniques, allowed us to use it as a standard. Therefore, we were able to unequivocally determine its retention time (RT) and MS fragmentation pattern and to subsequently compare such data with those observed for OA. Initially, OA (**1**) and 19-*epi*-OA (**2**) were analysed separately using an adequate liquid chromatography method coupled with ionization mass spectrometry that was developed for previously reported metabolites from *P. belizeanum* [16]. Afterwards, a mixture containing 2.5 ng/μL of 19-*epi*-OA (**2**) together with OA (**1**) was analysed both in positive and negative modes (Figure 2).

Our results showed that, under such conditions, 19-*epi*-OA eluted at RT 6.46 min, slightly earlier than OA, which shows a RT of 7.36 min. Therefore our data is consistent with those previously reported by Quilliam in 1995 and Vale in 2006 [12, 13], where the presence of a new toxin (named iso-OA by Vale) with shorter retention time than OA was detected. However, the authors in those articles did not give further structural details.

Under our experimental conditions, ionization in positive mode gave mainly the [M+NH_4_]^+^ ion at *m*/*z* 822 for both toxins, together with small amounts of [M+Na]^+^ and [M+H]^+^ ions (Figure 2 C and E). Ionization in negative mode generated the [M−H]^−^ ion for both toxins at *m*/*z* 803, as well (Figure 2 D and F). Thus, even though the generation of a single molecule-related ion should give an easier to understand chromatogram when negative ionization mode is used, it should be noted that the intensity of the peak detected in negative ionization mode is lower than in positive ionization mode.

**Figure 3:**
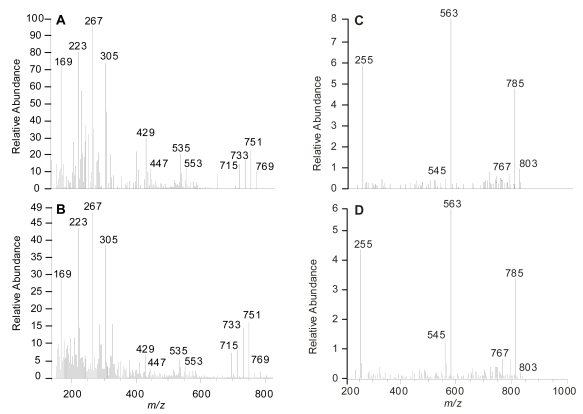
MS/MS product ion spectra of the positive ion at *m*/*z* 822 [M+NH_4_]^+^ for (A) okadaic acid and (B) 19-*epi*-okadaic acid; and in the negative ion mode at *m*/*z* 803 [M−H]^−^ for (C) okadaic acid and (D) 19-*epi*-okadaic acid. Collision energy was 40 eV. All *m*/*z* values were rounded down.

The comparative MS/MS fragmentation in positive ionization mode showed, for both OA (**1**) (Figure 3A) and 19-*epi*-OA (**2**) (Figure 3B), predominant ion peaks corresponding to the ion adducts at *m*/*z* 822 [M+NH_4_]^+^ reported for OA (**1**). The consecutive losses of H_2_O molecules produced ions at *m*/*z* 769, 751, 733 and 715, as well as several characteristic fragments such as *m*/*z* 553, 535, 429, 305, 267, 223 and 169 [12]. Furthermore, from the negative ionization mode study for OA (Fig. 3C) and 19-*epi*-OA (**2**) (Figure 3D) no significant differences could be found in the fragmentation profile. These resemblances could be summarized by the presence of the predominant peak sequence with ions at *m*/*z* 803 [M−H]^−^ →785→563→255 for both molecules.

The LC-MS/MS data obtained by us using both, positive and negative ionization mode, indicated that OA and its isomer 19-*epi*-OA show easily distinguishable RTs in LC using the experimental conditions proposed by us, but almost identical fragmentation patterns in MS. It is well know that LC-MS is an important tool for identifying several types of marine toxins. However, it is clear that in some instances, and this work is a good example, previous isolation and full structure elucidation of the new toxins, such as 19-*epi*-OA, is indeed indispensable, as it is necessary to have the reference toxins to be able to use them as unequivocal standards [17]. Therefore in this work, using the data extracted from our reference material, RT and MS/MS fragmentation patterns were obtained for 19-*epi*-OA, opening the possibility to detect a new DSP toxin congener’s routinely.

## 3. Experimental Section

### 3.1 Toxin source

The dinoflagellate *Prorocentrum belizeanum*, strain PBMA01, was obtained from the culture collection of phytoplankton at the Centro Oceanográfico in Vigo (CCVIEO) by courtesy of Mr. Santiago Fraga. This strain was originally isolated from a coral reef of La Reunion Island, Indian Ocean, France.

### 3.2 Preparation of batch algal cultures

Large scale cultures of the *P. belizeanum* strain were grown in 80 L tanks containing 40 L of sea water enriched with Guillard K medium at 23 ±1 °C under a 16:8 light:dark cycle. Cell cultures were scaled up to a final volume of 1,020 L and incubated statically for 5 weeks until a cell density of 20,000 cells/mL.

### 3.3 Toxins extraction and isolation

Cell cultures were harvested by discarding the supernatant and afterwards by centrifugation at 3,700 × *g.* Toxins were extracted by sonication of the cells with 1L of acetone three times and afterwards with 1L methanol another three times. The solvents were then evaporated and the resulting extracts combined. Toxin purification was achieved by LC using, as a first step, gel filtration on a Sephadex LH-20 column eluted with a mixture of chloroform-methanol-*n*-hexane (1:1:2). Next a medium-pressure reversed-phase Lobar LiChroprep RP-18 column was eluted with acetonitrile-water (1:1) and methanol-water (3:2). Toxin-containing fractions were pooled and subjected to a HPLC final purification on an XTerra column using an isocratic elution with methanol-water (7:3) to obtain the previously reported toxins OA (**1**), 7′-hydroxymethyl-2′-methylene-octa-4′,7′-dienyl okadaate and DTX-5c [16] as well as 19-*epi*-OA (**2**) [15], which has not been characterized by LC-MS previously.

### 3.4. LC-MS analysis

Chromatographic separations were carried out on a Waters XBridge C-18 5μ (150 × 2.1 mm) HPLC column thermostatized at 30 °C in a column oven. A mixture of two mobile phases, (A) ammonium acetate 2 mM, pH 5.8 and (B) methanol were used as eluents under the following chromatographic conditions: the initial eluent composition 60 % of B (6 min), followed by a flash ramping (7 min) to 70 % B and slow ramping (28 min) to 80 % B and a flash ramping (30 min) to 100 % B, maintaining this condition at 32 min and then returning to initial condition at 35 min. The flow rate was 200 μL/min and a volume of 5 μL of a 2.5 ng/μL solution of OA (**1**) and 19-*epi*-OA (**2**) in methanol was injected into the LC-MS system.

LC-MS measurements were performed using an ion trap mass spectrometer, Thermo Finnigan LCQ-Advantage, equipped with an atmospheric pressure ionization source and an electrospray ionization interface (ESI). ESI was performed using a 5.5 kV spray voltage at 200 °C capillary temperature and a flow of 4 L/min of sheath gas and 20 mL/min of auxiliary gas. Full scan spectra were acquired both, in negative and positive ion modes in the mass range *m*/*z* 220–1000. Selected ion chromatograms for both OA (**1**) and 19-*epi*-OA (**2**) were performed in negative ion mode at *m*/*z* 803 [M−H]^−^, and in positive ion mode at *m*/*z* 805 [M+H]^+^; 822 [M+NH4]^+^ and 827 [M+Na]^+^.

LC-MS/MS analysis was performed in a triple-quadruple mass spectrometer, Thermo Finnigan TSQ-Quantum Discovery, equipped with an electrospray ionization interface (ESI). ESI was performed using a 4 kV spray voltage at 300 °C capillary temperature and a flow of 30 mL/min for sheath gas and 5 mL/min for auxiliary gas. Full scan spectra were collected from *m*/*z* 150 to 900, in both negative and positive ion modes. Applying a supplementary voltage (Collision Energy, CE) of 40 eV on the precursor ions at *m*/*z* 803 [M−H]^−^ and 822 [M+NH_4_]^+^.

## Figures and Tables

**Figure 1:**
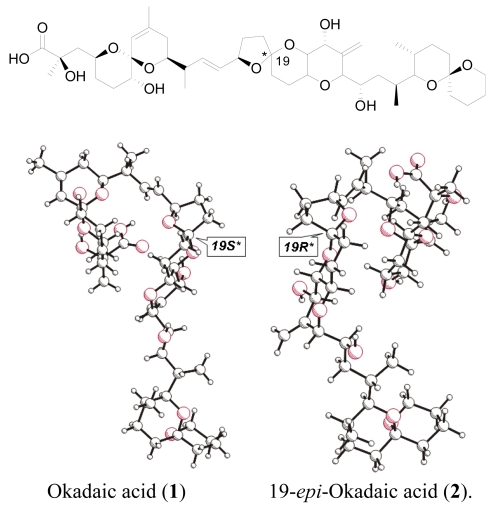
Planar and 3D structures for okadaic acid (**1**) and 19-*epi*-okadaic acid (**2**).
